# A dataset of chemical reaction pathways incorporating halogen chemistry

**DOI:** 10.1038/s41597-025-05944-3

**Published:** 2025-10-20

**Authors:** Minhyeok Lee, Jinyoung Jeong, Islambek Ashyrmamatov, Umit V. Ucak, Sunwoo Kim, Juyong Lee, Eunji Sim

**Affiliations:** 1 Department of Chemistry, Yonsei University, 50 Yonsei-ro, Seodaemun-gu, Seoul, 03722 Republic of Korea; 2 College of Pharmacy, Seoul National University, 1 Gwanak-ro, Gwanak-gu, Seoul, 08826 Republic of Korea; 3 Research Institute of Pharmaceutical Science, College of Pharmacy, Seoul National University, 1 Gwanak-ro, Gwanak-gu, Seoul, 08826 Republic of Korea; 4 Department of Molecular Medicine and Biopharmaceutical Sciences, Graduate School of Convergence Science and Technology, Seoul National University, 1 Gwanak-ro, Gwanak-gu, Seoul, 08826 Republic of Korea; 5 Arontier Co., 241, Gangnam-daero, Seocho-gu, Seoul, 06735 Republic of Korea

**Keywords:** Computational chemistry, Cheminformatics

## Abstract

Machine learning interatomic potentials (MLIPs) promise to revolutionize computational chemistry; however, their performance depends critically on the quality and diversity of the training data. Existing quantum chemical datasets predominantly focus on equilibrium structures and exhibit limited halogen coverage, despite halogens being present in approximately 25% of pharmaceuticals and numerous materials. We present Halo8, a comprehensive dataset that addresses this gap by systematically incorporating fluorine, chlorine, and bromine chemistry into reaction pathway sampling. Using our efficient multi-level computational workflow, which achieves a 110-fold speedup over pure DFT approaches, Halo8 comprises approximately 20 million quantum chemical calculations from 19,000 unique reaction pathways. The dataset combines recalculated Transition1x reactions with new halogen-containing molecules from GDB-13, employing systematic halogen substitution to maximize chemical diversity. All calculations were performed at the *ω*B97X-3c level, providing accurate energies, forces, dipole moments, and partial charges. Validation demonstrates that Halo8 captures diverse structural distortions and chemical environments essential for reactive systems, serving as a valuable resource for training MLIPs applicable to pharmaceutical discovery, materials design, and catalysis.

## Background & Summary

Machine learning interatomic potentials (MLIPs) are transforming computational chemistry by combining the accuracy of quantum mechanical methods with the speed of classical force fields. These models learn from quantum chemical data to predict molecular energies and forces, enabling simulations of chemical processes at unprecedented scales^1–3^. However, the performance of MLIPs critically depends on the quality and diversity of their training data. While significant progress has been made in developing quantum chemical datasets, most existing datasets focus on equilibrium structures or limited chemical spaces, constraining the transferability and applicability of trained models to complex chemical systems.

Halogen atoms play crucial roles across chemistry, from pharmaceutical drug design, where 25% of small-molecule drugs contain fluorine^4^, to materials science, where halogenated compounds serve as key building blocks for organic electronics and polymers^5,6^. Despite their importance, halogen representation in quantum chemical datasets remains limited. The QM series^7–10^ laid the groundwork for MLIP development, focusing primarily on H, C, N, O, and F atoms, with fluorine appearing in less than 1% of QM7-X structures. The ANI series^11–16^ expanded this foundation with extensive conformational sampling, and ANI-2x notably included both fluorine and chlorine atoms, although these datasets emphasize equilibrium and near-equilibrium configurations rather than reactive processes. Transition1x^17^ marked a significant advance as the first large-scale dataset for chemical reactions, focusing on C, N, and O heavy atoms without including halogens. The absence of halogen chemistry in reaction pathway datasets presents challenges for MLIPs when modeling halogen-specific reactive phenomena, such as halogen bonding in transition states, changes in polarizability during bond breaking, and the unique mechanistic patterns of halogenated compounds. This gap motivates the need for dedicated halogen-focused reaction pathway data.

**Fig. 1:**
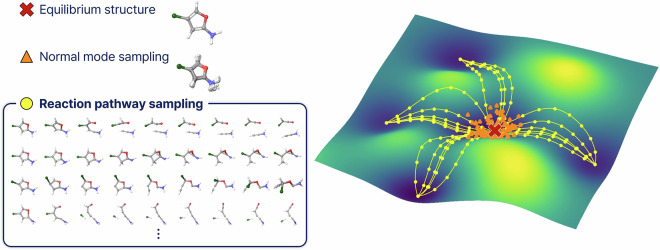
Sampling strategies for chemical reaction space. (Left) Example molecular structures demonstrating three approaches: single equilibrium geometry, overlaid structures from normal mode sampling (NMS) showing local perturbations, and structures from reaction pathway sampling (RPS) spanning multiple reaction channels. (Right) Conceptual potential energy surface illustrating how RPS explores diverse reactive regions beyond the local equilibrium well.

Our recently developed reaction pathway sampling (RPS) method^18^, which builds upon Transition1x, addresses the limitations of traditional approaches to generating training data. As illustrated in Fig. 1, equilibrium sampling yields only local minima and normal mode sampling (NMS)^10,11^ captures their local perturbations in the same energy basin, which limits the coverage of conformational space. In contrast, RPS systematically explores potential energy surfaces by connecting reactants to products, capturing structures along minimum energy pathways as well as intermediate configurations encountered during pathway optimization, including transition states, reactive intermediates, and bond-breaking/forming regions absent from equilibrium-focused datasets. While the recent OMol25 dataset^19^ interpolates between known reactant-product pairs, Halo8 discovers entirely new reaction pathways through automated exploration, focusing on underrepresented halogen chemistry to provide the out-of-distribution structures critical for training reactive MLIPs. Our multi-level protocol achieved a 110-fold acceleration over density functional theory (DFT)-only workflows, making large-scale reaction sampling practical and feasible. This efficient approach provides the diverse structural and energetic data necessary for training MLIPs that can describe dynamic chemical processes.

Here, we present Halo8, a comprehensive transition pathway dataset that systematically incorporates halogen chemistry using the RPS framework. The dataset augments recalculated Transition1x reactions^17,20^ with new halogen-containing molecules, introducing fluorine, chlorine, and bromine across diverse chemical environments. Through systematic halogen substitution and reaction discovery, Halo8 comprises approximately 20 million quantum chemical calculations derived from about 19,000 unique reaction pathways. The dataset includes structures with energies, forces, dipole moments, partial charges, and other properties, all computed at the *ω*B97X-3c level of theory^21^, a dispersion-corrected composite method with an optimized basis set. By combining the chemical diversity of halogen chemistry with the configurational diversity of RPS, Halo8 enables the training of MLIPs that can accurately model both equilibrium properties and reactive processes involving halogens, addressing a critical gap in current machine learning approaches to computational chemistry.

## Methods

### Reactant Selection

**Fig. 2:**
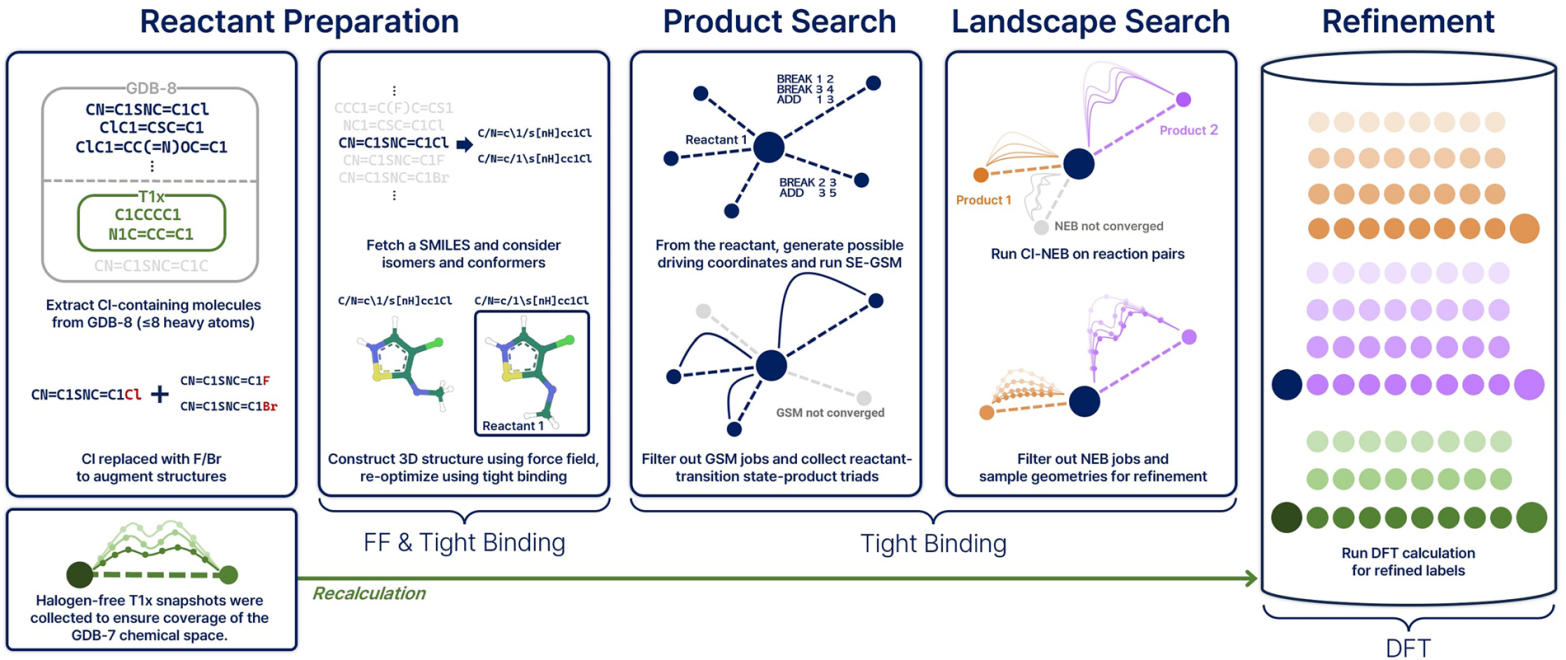
Halo8 dataset generation workflow. Molecule selection combines recalculated Transition1x (T1x) species^17^ (up to 7 heavy atoms, C/N/O) with new halogen-containing molecules from GDB-8 (the subset of GDB-13^22^ containing molecules with up to 8 heavy atoms), followed by systematic Cl → F/Br substitutions. Each molecule then follows the established pipeline: reactant preparation using a force field and GFN2-xTB optimization, product search via the single-ended growing string method (SE-GSM) to identify reaction products and transition states, landscape exploration using the nudged elastic band (NEB) method to sample reaction pathways, and final refinement with high-level DFT calculations.

The Halo8 dataset construction began with the systematic selection of molecules from the GDB-13 dataset^22^, as outlined in Fig. 2. For consistency with existing datasets, we first recalculated all Transition1x molecules sourced from GDB-13 subsets containing up to 7 heavy atoms (C, N, and O only). We incorporated halogen chemistry by extracting chlorine-containing molecules from GDB-8, a subset of GDB-13 that contains molecules with up to 8 heavy atoms. Each chlorine atom was then systematically substituted with fluorine and bromine, generating two additional molecules from a parent molecule to expand the halogen chemical diversity. All molecules underwent comprehensive structure preparation: RDKit^23^ was employed for stereoisomer enumeration and canonical SMILES^24^ generation, followed by 3D coordinate generation using the MMFF94 force field^25^ and OpenBabel^26^ with conformer searching to ensure diverse starting geometries. Final structures were optimized using GFN2-xTB^27^, providing consistent molecular geometries across the entire dataset for subsequent reaction pathway calculations.

### Computational Workflow

Our computational pipeline Dandelion, established in our previous work^18^, processes each molecule through systematic reaction discovery and characterization. In the reactant preparation stage, molecules undergo the geometry optimization described above. The product search via single-ended growing string method (SE-GSM) explores possible bond rearrangements from the reactant, with driving coordinates generated automatically^20,28,29^. Successfully identified reaction pathways, then proceed to landscape exploration using nudged elastic band (NEB) calculations with climbing image^30,31^ for better transition state location. We applied filtering criteria to ensure chemical validity, excluding trivial pathways with strictly uphill energy trajectories, negligible energy variations, or repetitive structures. To avoid redundancy in structural data, a new band was sampled only when the cumulative sum of Fmax exceeded 0.1 eV/Å since the last inclusion. Additionally, pathways were required to exhibit proper transition state characteristics, namely a single imaginary frequency. The final refinement stage performs single-point DFT calculations on selected structures along each pathway. This multi-level approach, utilizing xTB for initial sampling, dramatically reduces computational cost while maintaining chemical accuracy, as validated in our previous work, which shows a 110-fold speedup over pure DFT-based workflows. The complete pipeline, Dandelion, enables efficient exploration of the reaction space while maintaining the chemical accuracy necessary for MLIP training.

### DFT Method Selection

**Fig. 3:**
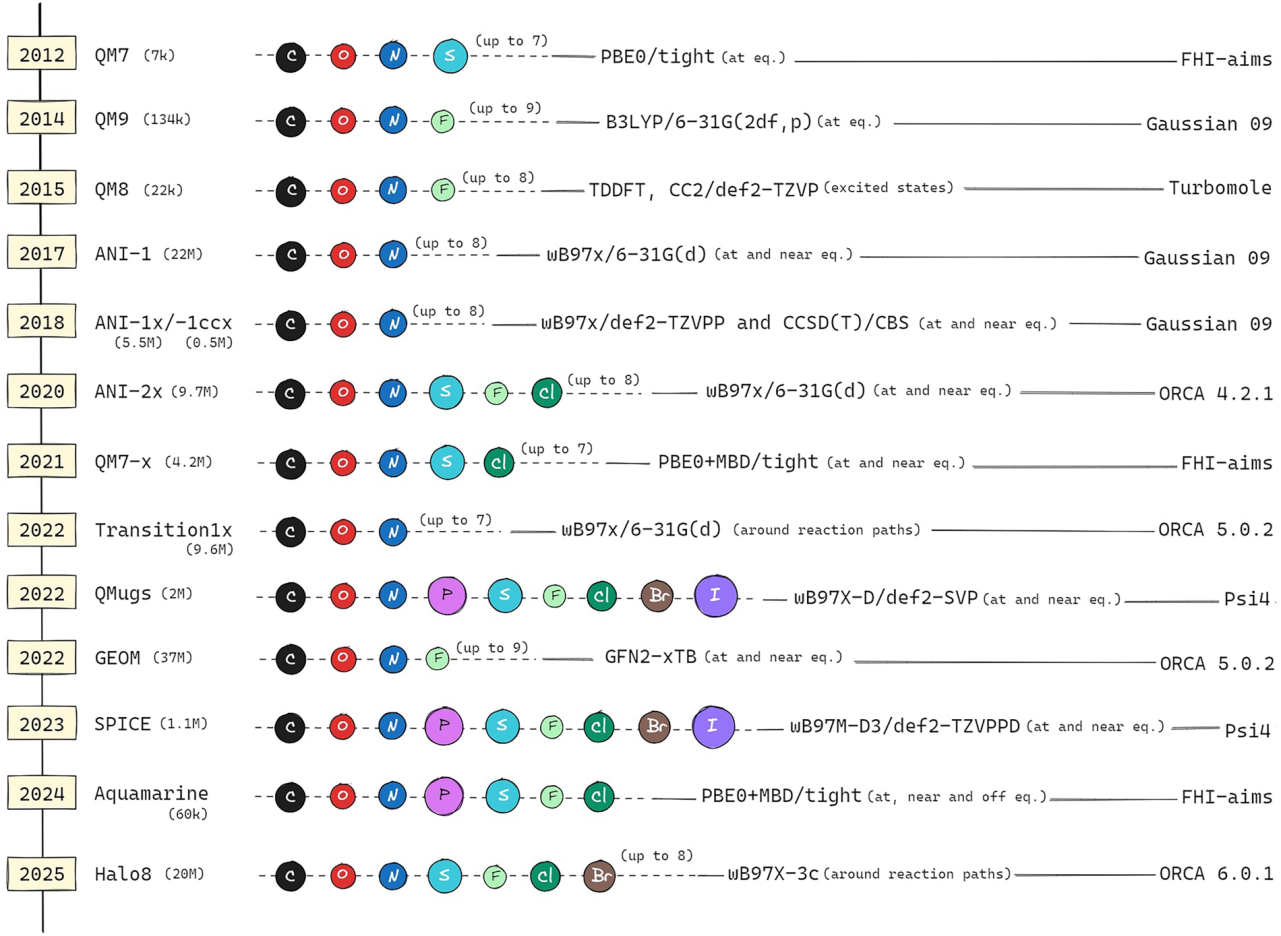
Timeline of quantum chemistry datasets for organic molecules. Chronological development of major datasets, including QM-series^7– 10^, ANI-series^13– 16^, as well as more recent large-scale efforts such as Transition1x^17^, QMugs^46^, GEOM^47^, SPICE^48^, and Aquamarine^49^. It highlights their size, elemental coverage, levels of theory, and software implementations, with emphasis on sampling goals.

**Fig. 4:**
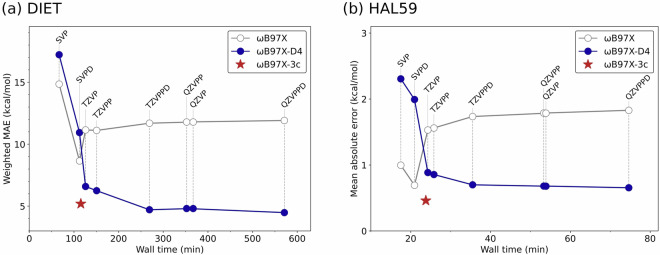
Computational cost-accuracy analysis for DFT method selection. (**a**) Weighted mean absolute error (MAE) on DIET benchmark^34^ versus wall time for variants of *ω*B97X with def2-series basis sets. (**b**) Mean absolute error on HAL59 dataset^35^. Comparison of *ω*B97X (gray), the dispersion-corrected variant *ω*B97X-D4 (blue), and the composite *ω*B97X-3c (red star) shows *ω*B97X-3c provides optimal cost-accuracy balance for dataset generation. All calculations were performed using 32 processors (two Intel(R) Xeon(R) Gold 6142 CPU @ 2.60GHz).

The timeline of quantum chemistry datasets (Fig. 3) illustrates the chronological evolution of datasets from early QM-series to recent large-scale efforts, each varying in elemental coverage, levels of theory, software implementations, and sampling strategies. Despite claims of compatibility between datasets using nominally identical levels of theory, differences in software implementations and versions lead to systematic discrepancies in computed properties, necessitating the recalculation of all Transition1x structures to ensure consistency within the Halo8 dataset. While the widely used combination of *ω*B97X/6-31G(d)^32,33^ is computationally efficient, this level has been proven to be insufficient for capturing dispersion interactions and polarizability effects, which are crucial for halogen-containing systems. Large basis sets are essential for accurately describing non-covalent interactions and electronic structure; however, computational constraints render extensive basis sets impractical for generating large-scale datasets. To identify an optimal method, we benchmarked various combinations on the DIET test set^34^ (Fig. 4a), a representative subset of the comprehensive GMTKN55 database^35^ that evaluates diverse chemical interactions including barrier heights, atomization energies, conformational energies, etc. The weighted mean absolute error (MAE) metric was used to normalize errors across molecules of different sizes and energy scales, enabling fair comparison. Performance was also evaluated on the HAL59 subset (Fig. 4b) from GMTKN55, which focuses on halogen dimer interactions, showing that *ω*B97X-3c delivers consistent accuracy for halogen-containing systems (F, Cl, Br), with similar trends observed across the broader organic molecular set. Notably, the *ω*B97X/6-31G(d) level employed for Transition1x showed unacceptably high weighted MAEs of 15.2 kcal/mol on DIET, with some DIET entries and most HAL59 entries unable to be calculated due to basis set limitations for heavier elements. While *ω*B97X-D4^36^ with the largest def2-QZVPPD basis set^37^ achieved the best accuracy (4.5 kcal/mol weighted MAE), the computational cost of 571 minutes per calculation rendered it infeasible for generating millions of data points. The *ω*B97X-3c composite method^21^ emerged as the optimal compromise, achieving 5.2 kcal/mol accuracy—comparable to quadruple-zeta quality—while requiring only 115 minutes per calculation, a five-fold speedup compared to the quadruple-zeta level. This method incorporates D4 dispersion corrections^38^ and utilizes a specially optimized basis set, providing an accurate treatment of molecular interactions at a manageable computational cost. All DFT calculations were performed using ORCA 6.0.1^39,40^ with the simple command !wB97X-3c notrah nososcf, where the notrah and nososcf keywords ensure consistent use of standard DIIS for SCF convergence. We note that the earlier version^41^ contained a bug in which the computed forces failed to sum to zero.

## Data Records

The Halo8 dataset is publicly available on Zenodo^42^. A readme file containing instructions on how to access the data stored in Halo8 is also available. The data are organized in the ASE (Atomic Simulation Environment) dataset format^43^, providing efficient storage and retrieval of molecular structures along with their associated properties. The dataset comprises approximately 20 million individual structures derived from about 19,000 unique reaction pathways, with each path containing  ≈1,000 structural snapshots along the reaction coordinate. Table 1 provides a detailed breakdown of the dataset composition: halogen-containing molecules account for approximately 10.7 million structures (3.8M with fluorine, 3.7M with chlorine, and 3.1M with bromine) from 9,341 reactions, while recalculated Transition1x molecules contribute 9.4 million structures from 9,835 reactions. The distribution spans molecules containing 3 to 8 heavy atoms, with the number of structures becoming larger due to the exponential growth in chemical diversity with molecular size. Each structure entry contains comprehensive quantum chemical data calculated at the *ω*B97X-3c level, as detailed in Table 2. Properties include atomic coordinates, energies, forces, frontier orbital energies, partial charges, dipole moments, and energy decomposition. Energies are reported in eV for machine learning conventions, while charges and dipole moments follow quantum chemistry conventions in atomic units.

**Table 1:** Summary of the Halo8 dataset composition.

Dataset component	Element	Reactant formulas	Total reactions	Number of structures by heavy atom count						Total structures
				3	4	5	6	7	8	
Halogen	F	38	3,317	—	—	—	23,342	219,972	3,596,240	3,839,554
	Cl	38	3,247	—	—	—	23,816	236,624	3,469,662	3,730,102
	Br	38	2,777	—	—	—	9,806	179,930	2,947,346	3,137,082
Transition1x		171	9,835	7,868	87,784	501,288	3,985,318	4,827,292	—	9,409,550
Total (Halo8)		285	19,176	7,868	87,784	501,288	4,042,282	5,463,818	10,013,248	20,116,288

**Table 2:** Data records for each structure in the Halo8 dataset.

Label	Size	Units	Type
Coordinates	N×3	Å	array
Energy	1	eV	float
Force	N×3	eV/Å	array
HOMO level	1	eV	float
LUMO level	1	eV	float
Mulliken charges	N×1	a.u.	array
Löwdin charges	N×1	a.u.	array
Dipole moment	1×3	a.u.	array
Nuclear repulsion energy	1	eV	float
Electronic energy	1	eV	float
One electron energy	1	eV	float
Two electron energy	1	eV	float
Exchange energy	1	eV	float
Correlation energy	1	eV	float
Dispersion correction	1	eV	float

## Technical Validation

**Fig. 5:**
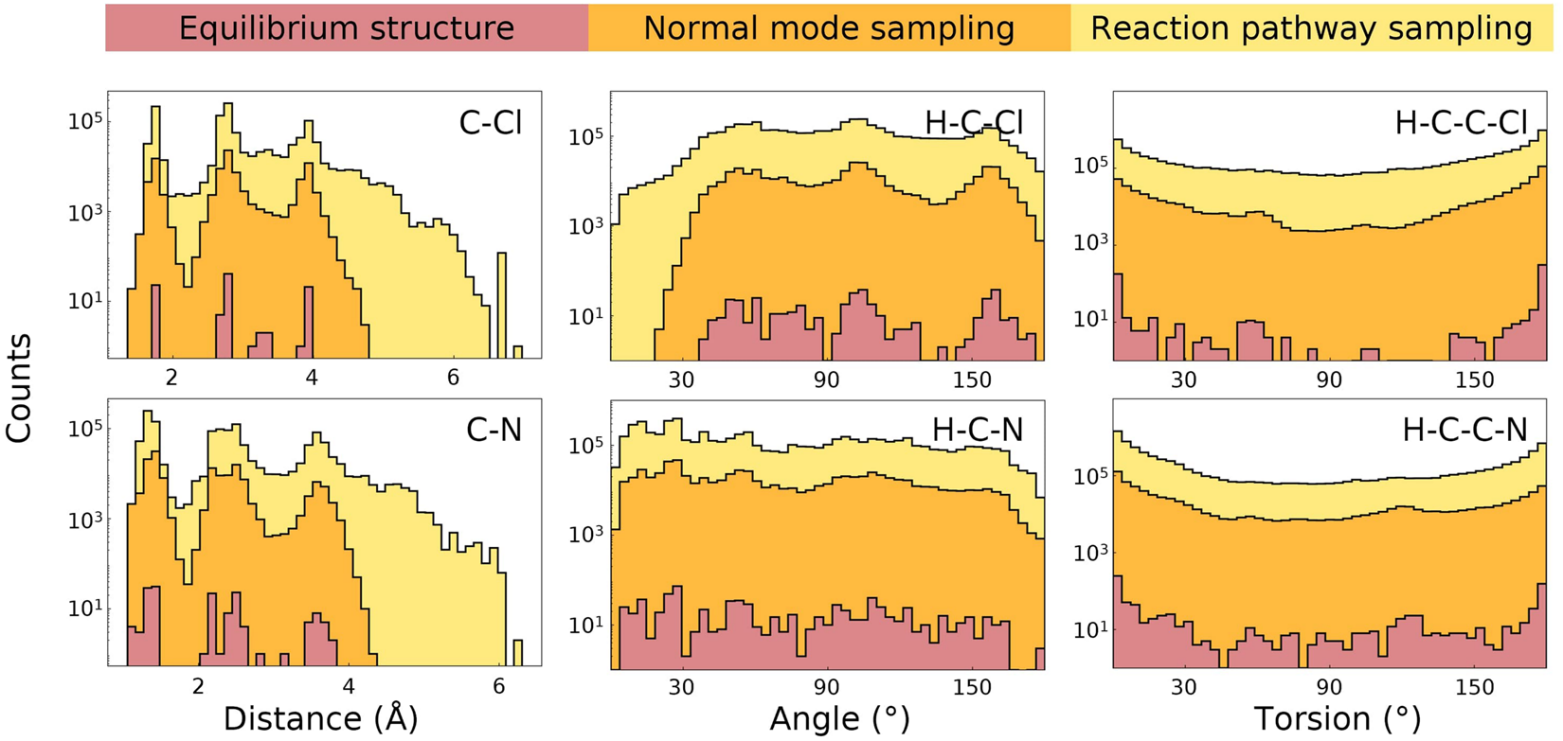
Structural diversity across sampling methods. Distributions of distances, angles, and torsions from the same starting structures comparing equilibrium geometry, normal mode sampling (NMS), and reaction pathway sampling (RPS), showing progressively expanded configurational space coverage.

The structural diversity of the Halo8 dataset was assessed by analyzing geometric parameter distributions across sampled configurations starting from identical equilibrium structures. Figure 5 demonstrates the progressively expanded coverage achieved through different sampling methods. For bond distances, equilibrium structures exhibit narrow peaks at typical bond lengths. NMS broadens these distributions slightly through thermal perturbations, while RPS captures distances extending to 7Å, including stretched bonds during dissociation and compressed geometries near transition states. Bond angle and torsion distributions reveal a similar progression: equilibrium structures produce a single conformation, NMS explores limited local variations, and RPS samples the full angular and torsional space, capturing highly distorted configurations essential for chemical reactions. This comprehensive geometric coverage ensures that MLIPs trained on Halo8 can accurately predict properties across the full range of molecular distortions encountered during chemical reactions.

**Fig. 6:**
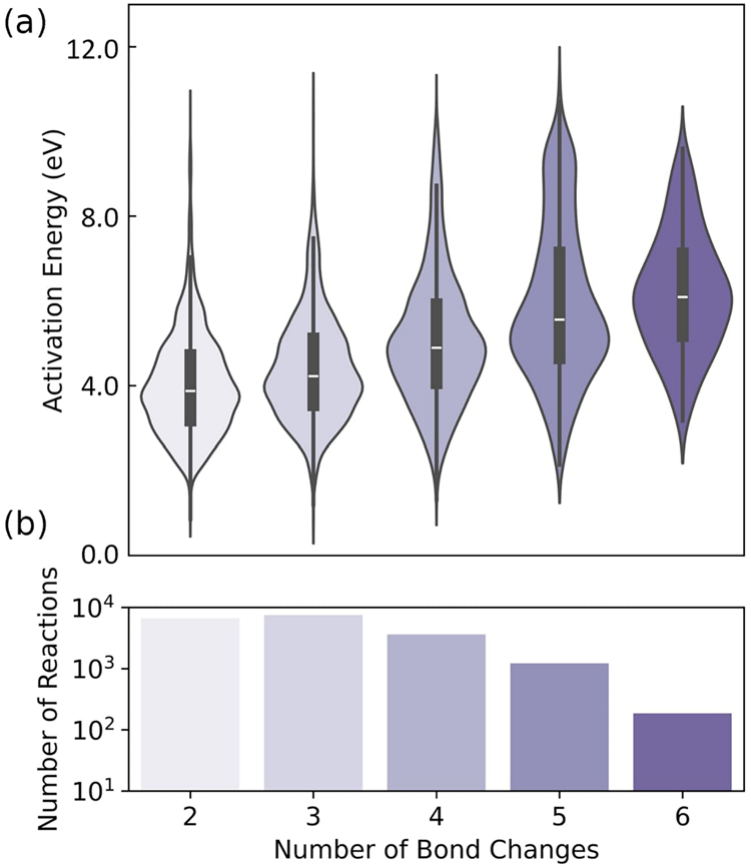
Reaction diversity in the Halo8 dataset. The dataset captures diverse reaction pathways, including high-energy transition states, which are essential for mapping reactive potential energy surfaces. (**a**) Distributions of activation energies categorized by the number of bond rearrangements. (**b**) The number of reactions classified by bond change complexity.

The dataset’s reaction diversity was quantified through an analysis of reaction energetics. Figure 6 presents activation energy distributions and reaction complexity, showing that Halo8 captures reactions involving 2 to 6 simultaneous bond changes. The activation energies increase systematically with reaction complexity, spanning from typical organic reaction barriers to high-energy processes requiring multiple bond rearrangements. The histogram shows that reactions with 2-3 bond changes are most prevalent, while more complex reactions involving 4-6 bond changes capture rarer but chemically important high-energy processes.

**Fig. 7:**
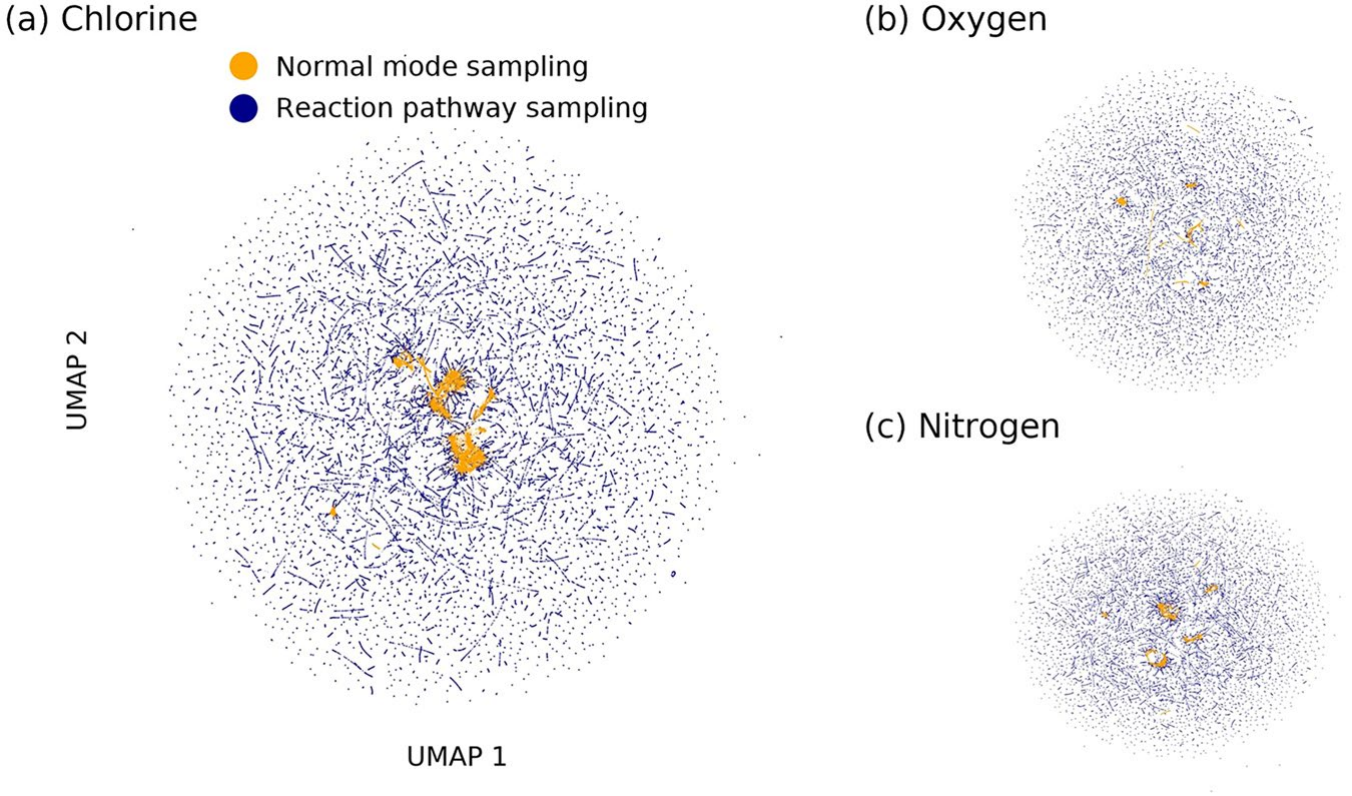
Chemical environment diversity captured by different sampling methods. UMAP visualization of MACE features showing (**a**) chlorine, (**b**) oxygen, and (**c**) nitrogen atoms from normal mode sampling (NMS, orange) clustered in feature space versus broadly distributed reaction pathway sampling (RPS, blue).

To validate chemical diversity at the atomic level, we analyzed local atomic environments using MACE model-learned representations^44^. Figure 7 shows the uniform manifold approximation and projection (UMAP) projections^45^ of atomic feature vectors for atoms sampled from identical reactant molecules using different sampling methods. NMS produces clustered features indicating limited environmental diversity, while RPS-sampled atoms distribute broadly across feature space, reflecting exposure to diverse bonding partners and electronic environments. This enhanced diversity is crucial for the transferability of MLIP. Models trained on near-equilibrium molecules must extrapolate when encountering reactive configurations, whereas the comprehensive coverage from RPS enables accurate interpolation across varied chemical contexts^17,18^.

## Data Availability

The dataset is available at 10.5281/zenodo.16737590.

## Code availability

The Dandelion codes^18^ used in this work are available at https://github.com/mhyeok1/dand.
