# Publisher Correction: A dataset of chemical reaction pathways incorporating halogen chemistry

**DOI:** 10.1038/s41597-026-06574-z

**Published:** 2026-01-15

**Authors:** Minhyeok Lee, Jinyoung Jeong, Islambek Ashyrmamatov, Umit V. Ucak, Sunwoo Kim, Juyong Lee, Eunji Sim

**Affiliations:** 1 Department of Chemistry, Yonsei University, 50 Yonsei-ro, Seodaemun-gu, Seoul, 03722 Republic of Korea; 2 College of Pharmacy, Seoul National University, 1 Gwanak-ro, Gwanak-gu, Seoul, 08826 Republic of Korea; 3 Research Institute of Pharmaceutical Science, College of Pharmacy, Seoul National University, 1 Gwanak-ro, Gwanak-gu, Seoul, 08826 Republic of Korea; 4 Department of Molecular Medicine and Biopharmaceutical Sciences, Graduate School of Convergence Science and Technology, Seoul National University, 1 Gwanak-ro, Gwanak-gu, Seoul, 08826 Republic of Korea; 5 Arontier Co., 241, Gangnam-daero, Seocho-gu, Seoul, 06735 Republic of Korea

**Keywords:** Computational chemistry, Cheminformatics

Correction to: *Scientific Data* 10.1038/s41597-025-05944-3, published online 20 October 2025

In this article the author’s name Umit V. Ucak was incorrectly written as Umit V. Ucaxk. The original article has been corrected.

